# LearningRx Cognitive Training for Workplace Self-Efficacy in Adults with Post-COVID-19 Brain Fog: A Mixed-Methods Pilot Study

**DOI:** 10.3390/brainsci16040410

**Published:** 2026-04-11

**Authors:** Amy Lawson Moore, Edward J. Jedlicka, James C. Patterson, Christina R. Ledbetter

**Affiliations:** 1 Department of Psychology, Gibson Institute of Cognitive Research, Colorado Springs, CO 80921, USA; amoore@gibsonresearch.org (A.L.M.); e.jedlicka@gibsonresearchinstitute.com (E.J.J.); 2 Department of Psychiatry and Behavioral Medicine (Ret.), LSU Health Shreveport, Shreveport, LA 71103, USA; 3 Department of Neurosurgery, LSU Health Shreveport, Shreveport, LA 71103, USA

**Keywords:** cognitive training, cognitive rehabilitation, cognition, memory, post-COVID-19 syndrome, long COVID, neuroplasticity, attention, focus

## Abstract

Background/Objectives: Cognitive dysfunction, or “brain fog”, following COVID-19 viral infection is strongly associated with diminished work capacity which disproportionality affects working-age adults. This study examined an existing method of cognitive rehabilitation training applied to adults struggling with workplace functioning and self-efficacy due to post-COVID-19 brain fog. Methods: Nine adults with post-COVID-19 cognitive dysfunction participated in this single arm pilot trial of a severity-adaptive cognitive training program. The participants completed 45–90 h of clinician-delivered cognitive training exercises delivered remotely in 60- to 90-min sessions, two or three times per week. The primary outcome measure was overall workplace self-efficacy with subskills of perceived workplace functioning, perception of cognitive functioning, and perception of home functioning assessed through pre and post surveys and qualitative interviews. The secondary outcome was cognitive function operationalized by an IQ score administered before and after the intervention. Results: The participants achieved significant improvements in workplace self-efficacy and cognition following cognitive training. The main qualitative themes of self-reported improvements were in executive function, health and energy, daily living activities, productivity, and socioemotional functioning. A cross-case synthesis of pre-intervention struggles, and post-intervention improvements revealed subthemes at work or school in cognitive processing and comprehension, memory, executive function, fatigue, emotional distress, confidence in work or academics, and work/academic performance impairment. As a group, the mean gain in IQ score was 10.5 points. Conclusions: This study adds to the growing body of literature examining the possibility of using cognitive rehabilitation for post-COVID-19 cognitive dysfunction impacting workplace self-efficacy and work functioning.

## 1. Introduction

Post-acute sequelae of COVID-19 (PASC), also referred to as post-acute COVID-19 syndrome (PACS) and long COVID, describes a heterogeneous constellation of persistent symptoms that can affect multiple organ systems following acute SARS-CoV-2 infection. Among these, neurological manifestations or neurological PASC (NC-PASC) are particularly prevalent and disabling, even in individuals who experience mild initial illness. Reported symptoms span cognitive, autonomic, and affective domains and commonly include cognitive dysfunction (“brain-fog”), impaired attention and memory, slower processing speed, fatigue, myalgia, headache, and dysautonomia [1,2,3]. The symptomatic profile of post-COVID-19 brain fog and cognitive dysfunction is still under debate with continuing discussions about classification, diagnostic criteria, and the etiologies which arguably complicate the management of the condition. Recent research illustrates the diversity of cognitive deficits that wax and wane in the months following infection including executive dysfunction (planning, executing tasks, multitasking), attention and concentration struggles, impaired memory (including verbal, visual, and working memory), visual processing deficits, and word finding problems [4,5]. Risk factors identified retrospectively have included female sex, fatigue, sleep problems, depression, rumination, and seeking in-person treatment during acute infection [6,7]. The “brain fog” experienced after COVID-19 has been described as similar to that experienced by patients undergoing chemotherapy as well as those with myalgic encephalomyelitis/chronic fatigue syndrome and mast cell activation syndrome [8]. Further, the downstream impacts of post-COVID-19 brain fog are also documented in recent research, including the impact on relationships, personal and professional identity [9], psychological well-being, and self-image [10]. The severity, prevalence, and combination of symptoms vary substantially across individuals, contributing to a marked clinical heterogeneity which complicates diagnosis, mechanistic interpretation, and treatment development. This is consistent with prior post-viral and post-infectious syndromes described after other acute infections [11]. Yet, there is not uniform diagnostic criteria or agreement on how severe symptoms must be for a diagnosis of post-COVID-19 brain fog.

A central controversy in the field concerns the pathophysiological basis of NC-PASC. While SARS-CoV-2 has been shown to access the central nervous system (CNS) through olfactory, vascular, and blood–brain barrier (BBB) associated routes [12,13,14,15,16], current evidence does not suggest widespread, persistent viral infection of the CNS as the primary driver of long-term cognitive symptoms [17,18]. Instead, converging lines of evidence suggest that NC-PASC arises from multiple interacting mechanisms, including chronic neuroinflammation, endothelial dysfunction, BBB disruption, microvascular injury, immune dysregulation, impaired neural plasticity, and mitochondrial dysfunction [19,20,21,22]. Recent molecular imaging work further suggests these upstream processes may converge at the synaptic level. This research shows evidence of widespread alterations in postsynaptic glutamatergic signaling associated with cognitive impairment in NC-PASC, consistent with diffuse network-level dysfunction rather than focal pathology [23]. These mechanisms may act synergistically to disrupt large-scale brain networks rather than producing focal deficits, offering one explanation for the diffuse and fluctuating cognitive symptoms reported by patients. Importantly, the diversity of the proposed mechanisms makes the likelihood of a single, mechanism-targeted therapy relatively low.

In parallel with mechanistic research, growing attention has been directed toward the functional consequences of post-COVID-19 cognitive impairment. Large-scale patient-led and longitudinal studies demonstrate that cognitive dysfunction is not only highly prevalent but also a major driver of disability in PASC. In a large multinational survey spanning over 3700 individuals with PASC, Davis et al. characterized the prevalence and functional consequences of cognitive dysfunction, reporting cognitive dysfunction and/or memory impairment in approximately 88% of respondents across all age groups. Further, they reported a strong association with impaired occupational functioning, noting over 86% of working participants reporting mild to severe difficulty performing their jobs [24]. Consistent with this, nearly half of the respondents required reduced work hours, and more than one-fifth were unable to work at the time of survey completion due to NC-PASC. These findings align with broader population-based studies showing that NC-PASC disproportionately affects working-age adults and contributes to significant societal and economic burdens [2,25,26].

Within this context, cognitive rehabilitation interventions have emerged as potentially promising, pragmatic approaches for addressing post-COVID-19 brain fog. However, outcomes have been inconsistent across interventions specifically targeting COVID-19-related cognitive dysfunction and return-to-work challenges. For example, Garcia-Molina et al. (2022) evaluated a post-COVID-19 rehabilitation program where 44.9% of participants remained unable to perform their workplace functions and 81.2% continued to experience difficulty with the activities of daily living following the completion of cognitive rehabilitation [27]. Similarly, in a 2025 scoping review of return-to-work interventions for individuals living with PASC, the few interventions classified as “promising” emphasized multidisciplinary, clinician-supported approaches, while self-directed digital rehabilitation programs showed limited evidence of effectiveness [28].

Nevertheless, evidence from longitudinal cohorts, systemic reviews, and early-phase intervention studies suggests that cognitive rehabilitation—particularly when adaptive, individualized, and multi-domain—can be associated with improvements in both objective cognitive performance and real-world functioning, including work participation and self-efficacy [29,30,31]. These findings are consistent with the broader cognitive neuroscience and rehabilitation literature demonstrating that repeated, targeted cognitive engagement can support recovery through experience-dependent neuroplasticity, even in the presence of ongoing neurobiological vulnerability [32,33,34].

Prior work by our group has demonstrated the feasibility and functional relevance of utilizing the LearningRx Brain Strong (formerly Brain Booster) and ThinkRx cognitive training programs in populations with neurodevelopmental and learning disorders, traumatic brain injury (TBI), and age-related cognitive decline. Across multiple studies, intervention participation was associated with significant improvements in cognitive performance, functional outcomes, and self-reported confidence in cognitive abilities with evidence of transfer beyond standardized test performance to everyday functioning [35,36,37,38,39,40,41,42]. These findings provide important precedent for applying a severity-tailored, multi-domain cognitive training approach to other heterogeneous neurological conditions characterized by diffuse network dysfunction, including NC-PASC.

Together, this body of work suggests that functionally oriented cognitive interventions, designed to accommodate individual variability and emphasize real-world relevance, may be particularly well suited to addressing post-COVID-19 cognitive sequelae. Accordingly, outcomes that capture confidence, competence, and perceived ability to function in occupational roles, such as workplace self-efficacy, may offer clinically meaningful indicators of recovery in early-phase intervention studies.

The primary aim of this study was to evaluate, using both quantitative and qualitative methods, pre–post changes in workplace self-efficacy following a clinically and commercially available, adaptive, severity-tailored cognitive training intervention in individuals with varying severity of post-COVID-19 brain fog. A secondary aim was to explore changes in objective cognitive function. Using a single-arm design and a small, intentionally heterogenous sample, analyses were exploratory, hypothesis-generating, and intended to identify preliminary signals of functional improvement and inform the design of future, controlled studies rather than to establish treatment efficacy [43,44].

## 2. Materials and Methods

### 2.1. Study Design and Implementation Considerations

The current study employed a prospective, single-arm, proof-of-concept interventional design to evaluate pre–post changes in workplace self-efficacy and cognitive performance following a clinically and commercially available, adaptive, severity-tailored cognitive training intervention in adults with post-COVID-19 brain fog.

All study procedures—including informed consent, screening, assessment, and intervention delivery—were conducted remotely using a secure videoconferencing platform (e.g., Zoom) with the exception of one participant who was tested in person at their request. This study was designed to protect participant privacy and maximize feasibility for working adults, reflecting real-world conditions under which the intervention would typically be delivered.

Initial recruitment occurred through local dissemination of study flyers and word-of-mouth outreach during the early phases of study planning when pre–post MRI imaging was under consideration. The subsequent sharing of recruitment materials on a long-COVID community message board generated immediate interest and enabled the enrollment of a geographically diverse cohort with heterogeneous disease severity.

Given that the intervention is commercially available and deliverable remotely without geographic constraints, geographical diversity was prioritized over the inclusion of neuroimaging as a secondary outcome measure. This decision supported feasibility while maintaining focus on functionally meaningful clinical outcomes.

### 2.2. Participants

The inclusion criteria consisted of adults aged ≥18 years with persistent subjective cognitive impairment (“brain fog”) lasting more than 60 days following COVID-19 who were working (including volunteer roles), enrolled in school, or unable to work or attend school due to COVID-19, and were able to provide informed consent and complete the study procedures. The exclusion criteria included non-English speakers, incarcerated individuals, those with a history of stroke, those pregnant at the time of consent, and individuals with unstable medical or psychiatric conditions. This last criterion was defined as any pre-existing condition still undergoing active treatment adjustments. It was included to minimize potential confounding effects as changes in these conditions could influence cognitive outcomes independent of post-COVID-19 symptomology.

### 2.3. Intervention

The cognitive training intervention used in this study was Brain Strong (formerly Brain Booster), a clinically and commercially available cognitive training program used in LearningRx cognitive training centers across the United States and in BrainRx centers globally. The training methodology has been extensively tested through a variety of research designs and is described in previous studies [35,36,37,38,39,40,41,42]; therefore, it is only briefly highlighted here.

**Figure 1:**
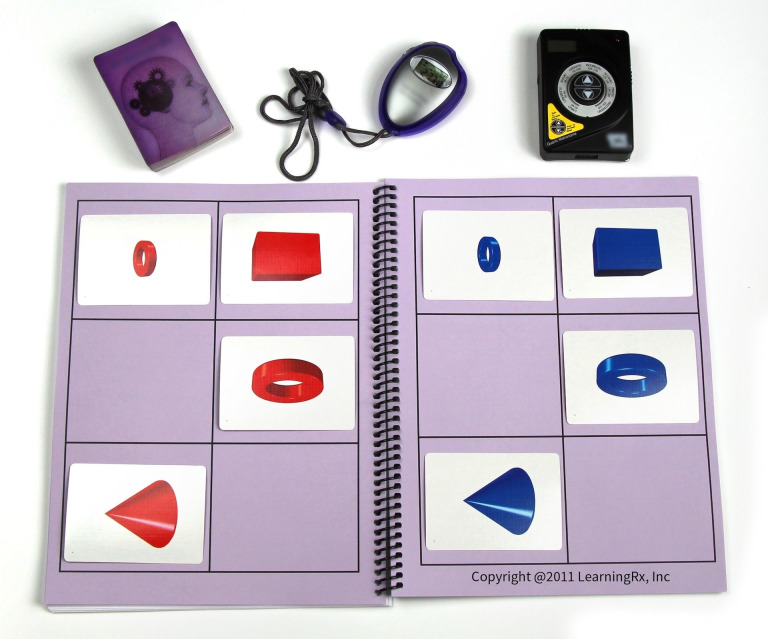
Example of a memory task from the Brain Strong program.

The Brain Strong intervention is an adult-focused version of the cognitive training methodology created by Dr. Ken Gibson. It consists of sixteen core mental exercises delivered by a cognitive trainer. Each training exercise has 8 to 12 variations sequenced in order of difficulty and complexity and designed to target multiple cognitive domains, including working memory, long-term memory, processing speed, fluid reasoning, visual processing, auditory processing, and attention. The training tasks are hands-on using a variety of manipulatives, cards, shapes, and workboards with grids and diagrams. Crucially, the tasks are structured to promote coordinated engagement of multiple cognitive domain networks within a single activity rather than an isolated practice of individual skills. For example, a memory task requiring the participant to reproduce patterns of shapes on a grid also engages visual processing, processing speed, and attention, particularly as the number of shapes increases and the time allotted for the task decreases. See Figure 1 for an illustration of this task. The trainer arranges cards in a pattern, allows the participant to study the pattern for only three seconds, covers the pattern, and cues the participant to recreate it from memory.

Each task is also paced using a metronome to increase the development of automaticity and to increase the intensity of the training session flow. Participants respond “on beat” to the metronome by counting aloud, giving answers to the actual task, or giving answers to an unrelated task as prompted by the trainer. For example, on the same memory task where the participant must reproduce a pattern of shapes from memory, he must also answer a math question or a logic question in the allotted time window.

The Brain Strong methodology was originally designed to be delivered in a clinic setting in person but has been adapted and rigorously tested in a remote environment as well. Notably, a non-inferiority study with 381 children and adults revealed no significant differences across cognitive skills between a group trained in person and a group trained remotely over Zoom [30]. In the current study, all the participants were trained remotely over Zoom. At the beginning of the study period, each participant was mailed an external webcam and all the hands-on materials needed for training. Training was delivered through one-on-one sessions with a participant’s assigned cognitive trainer who was trained and certified in the program’s delivery. The intervention was delivered using an adaptive, severity-tailored protocol with total training durations ranging from 45 to 90 training hours.

All participants began with a minimum training duration of 45 h. The participants attended two or three 60–90 min training sessions per week based on participant preference, tolerance, and schedule. Following completion of the initial 45 h, additional training hours were assigned in 15-h increments, determined through ongoing needs assessments and collaborative discussions involving the participant, the cognitive trainer, and the study director. This adaptive and flexible delivery model mirrors standard clinical practice in LearningRx and BrainRx centers and therefore, supports the ecological validity of the intervention. For example, intervention progress was carefully monitored using a web-based training management system. This method of managing the scope and sequence of training procedures enables dynamic assessment and progress monitoring in real-time. Every attempt at mastery was noted by the cognitive trainer in the training management system using an iPad on the trainer’s desk. By reviewing this training data, the cognitive trainer and study team could assess when a participant was not making progress on certain training tasks or on individual constructs (memory, attention, processing speed, etc.). Decision-making about the number of additional training sessions was both objective (using the training progress data) and subjective (based on a review of trainer session notes and clinical judgement). There was collaboration with the participant in these decisions as well. If the cognitive trainer and study team determined more training was needed, the participants were asked if they were interested in continuing beyond the initial training period, if they were motivated to continue, and if they had the capacity in their schedule to continue. Because of the iterative process involved in these protocols, doctoral level research team members with significant experience in the delivery and supervision of this complex intervention were included to ensure protocol fidelity, and all relevant relationships with the intervention developer were disclosed.

### 2.4. Assessment Measures and Data Collection

The primary outcome for this study was workplace self-efficacy. Two measures were used to assess the construct: a Workplace Self-Efficacy Survey and a semi-structured interview. The secondary outcome for this study was cognitive function, measured and operationalized by a standard IQ test. Data from all three measures were collected before and after the intervention.

#### 2.4.1. Workplace Self-Efficacy Survey

The Workplace Self-Efficacy Survey is a 29-item Likert-style functional rating scale with questions and response options on a scale from 1 (low functioning) to 10 (high functioning) developed by Christina Ledbetter at LSU specifically for the current study. The questions are divided into three categories: (1) perceived performance at work or school (20 items), (2) perceived cognitive functioning at work or school (6 items), and (3) perceived functioning at home (3 items). A composite score on overall workplace self-efficacy is also generated by adding the scores on categories 1 and 2, creating a total of four scales.

Appendix A.1 list the survey items. Each participant completed the survey twice: once at the beginning of the study and again after completing the intervention. When a participant was enrolled in the study and had completed their informed consent documents, the study director emailed the participant a link to the survey using RedCap [45,46], a secure web application for electronic data capture hosted at LSU and compliant with HIPAA and 21CFR Part 11. It is used by more than 8000 institutions for administering and collecting survey data.

After receiving the link, each participant was asked to rate themselves on each item as they recall functioning prior to getting COVID. Then, they were asked to rate how they perceive themselves to be functioning currently on each item (after getting COVID-19 but before starting the intervention). After the intervention, the participant was asked to rate their perceived functioning on each item again. To minimize expectancy effects and encourage realistic reporting, they could not see their original responses from the beginning of the study. In response to reviewer feedback regarding the lack of psychometric properties in our survey, we calculated the internal consistency reliability of the four scales with Cronbach’s alpha on the baseline data from Time 1. Three scales had strong internal consistency reliability: Overall Workplace Self-Efficacy (α=0.91), Workplace Performance (α=0.83), and Cognitive Functioning (α=0.84). The Home Functioning scale had low internal consistency (α=0.48), likely due to the small number of items on the scale (n = 3). Therefore, the Home Functioning questions are analyzed individually rather than as a composite score. Approaching this with item-level analysis will aid in exploratory insight without aggregating items that appear heterogenous.

#### 2.4.2. Qualitative Interviews

The second method for assessing the primary outcome of workplace self-efficacy was through pre and post semi-structured interviews. Each participant in the current study was interviewed two times: once at the beginning of the study and again upon completion of the intervention. The interviews were conducted by doctoral-level researchers in the fields of psychology and neuroscience using the items on the Workplace Self-Efficacy Survey as the interview guide. The interviewer asked the participant to comment on each of their ratings and to give an example of how the item impacts them at work or school or at home. For example, in the baseline interview, Item #3a Ability to maintain workspace order or neatness BEFORE COVID-19 served as a prompt for the question, “Give me an example of your ability to do that. What did that look like for you?” Then, the interviewer would ask for an example of how each ability changed AFTER getting COVID-19. Item #3b Ability to maintain workspace order or neatness AFTER COVID-19 served as a prompt for the question, “What does that look like for you after getting COVID. How did that ability change?” At the exit interview, the interviewer would ask the participant to comment on their ratings of each item after completing the intervention. For example, Item #3c Ability to maintain workspace order or neatness currently AFTER completing cognitive training served as a prompt for the question, “Tell me about your rating on this item. What does that look like for you now? What has changed since completing cognitive training?”

In addition to asking about the participant’s ratings on the survey, the interviewer also asked the participant to talk about any other changes they noticed since completing cognitive training. To encourage self-reflection, the interviewer was careful to use neutral terms such as “changes” versus “improvements” and asked general questions such as “What have you noticed about ______?” rather than asking leading questions such as “What improvements have you seen in ______?”

Following the interviews, the recordings were transcribed using Descript software v139.0.10. The transcripts were then downloaded for analysis.

#### 2.4.3. Cognitive Testing

The secondary outcome for the current study was change in overall cognition which we operationalized using an IQ score, or the General Intellectual Ability (GIA) composite score from the Woodcock Johnson IV (WJIV) Tests of Cognitive Abilities [47]. The GIA is a weighted composite of 7 subtests measuring the following constructs: fluid reasoning, short-term working memory, long-term memory, visual processing, auditory processing, processing speed, and prior knowledge. The GIA score has a median reliability of 0.97 across a normative sample [48]. The WJIV was administered either remotely or in person by a single doctoral level psychologist with extensive experience in both remote and in-person assessments. Administration procedures were standardized and followed the test publisher’s specific guidance for both in-person and remote assessments. Raw scores were uploaded into Riverside Score, the online scoring system for the assessment, and then the score report was downloaded for analysis.

### 2.5. Data Analysis

#### 2.5.1. Workplace Self-Efficacy Survey Statistical Analysis

The primary outcome for this study was to evaluate changes in perceptions of workplace self-efficacy for the participants. We first assessed this outcome using the scores on the Workplace Self-Efficacy Survey (WSES) completed by the participants. Statistical analysis was conducted using the General Linear Model (GLM) procedure in SPSS, Version 29. Specifically, we performed a within-group group repeated measures Multivariate Analysis of Variance (MANOVA) to determine whether the overall Workplace Self-Efficacy Score as well as ratings of perceived work performance and perceived cognitive functioning at work changed over three timepoints: before COVID-19, after getting COVID-19, and after cognitive training. A MANOVA allowed us to determine if a categorical independent variable has a significant effect on the multiple dependent continuous variables. For this analysis, we used Time as the independent variable and the three composite scores on the WSES as the dependent variable (with three timepoints). Partial eta squared was the metric we used for effect size, classified as small ($\eta_{p}^{2}$ = 0.01), medium ($\eta_{p}^{2}$ = 0.06), and large ($\eta_{p}^{2}$ ≥ 0.14). Univariate tests (repeated measures ANOVA) were extracted from the GLM to tell us where the differences lie. The significance threshold was set at *p* < 0.05 and a Bonferroni correction was used to control for multiple comparisons.

Because the three items on the Home Functioning scale had low internal consistency, we could not include them in the MANOVA with the other scales. Instead, we used nonparametric tests to analyze the three individual questions from the Home Functioning scale. Specifically, we chose the nonparametric alternative to ANOVA for repeated measures since it does not rely on dependent variables to be moderately correlated like MANOVA does. Therefore, using SPSS Version 29, we analyzed the three individual items using the Friedman test followed by pairwise Wilcoxon signed-rank tests with Bonferroni correction to identify specific differences between timepoints.

#### 2.5.2. Interview Data Analysis

We analyzed the interview data from pre- and post-intervention interviews using a codebook-style thematic analysis based flexibly and pragmatically on Braun and Clark’s 6 phase framework: familiarization, coding, theme development, review, definition, and write-up [49]. This method of analyzing interview data is the most common approach adopted by qualitative researchers with over 300,000 citations as of this writing. First, we familiarized ourselves with the transcripts by reading them multiple times. We took notes on initial patterns and recurring ideas specifically related to the topics in the interview guide which was based on the Workplace Self-Efficacy Survey.

Next, we generated an initial coding framework called a codebook based on the interview guide to ensure consistency. Then, we went line by line through the interview transcripts and coded the data using descriptors that captured key concepts from both the pre- and post-intervention interviews. We used sentences and phrases as our units of meaning. We tracked pre- versus post-intervention occurrences to facilitate a longitudinal comparison.

Then, we developed our themes. We created code-by-participant matrices and then put similar codes into clusters that addressed like areas of functioning to help develop potential themes and subthemes. We iteratively reviewed and compared the themes to the overall dataset to ensure they adequately represented the data, that they were clearly distinguished from one another, and that they represented both similarities and differences across participants and timepoints. We merged similar themes, split themes that were too complex, and discarded themes that did not match the data.

After developing and reviewing the themes, we clearly defined them and gave them clear and concise names. Finally, we conducted descriptive counts of how many participants reported data in each main theme to show pattern strength, and we selected representative examples for the write up. We also developed a cross-case synthesis matrix using pre-intervention subthemes to illustrate more detail in the participants’ experience of workplace self-efficacy over time.

#### 2.5.3. Cognitive Testing Data Analysis

The secondary outcome for this study was to quantify changes in cognition using IQ scores for the participants. We assessed this outcome using the General Intellectual Ability (GIA) composite scores on the Woodcock Johnson Tests of Cognitive Abilities (WJIV). Statistical analysis was conducted using the Compare Mean and Proportions procedure in SPSS, Version 29. Specifically, we performed a paired samples *t* test on pre-intervention and post-intervention standard scores to determine whether there was a significant change following the intervention. We selected a paired samples *t* test because it is specifically designed for studies where the same participants are measured twice. This test tells us if their mean change in IQ score is significantly different from zero. We report Cohen’s *d* as the metric for effect size classified as small (*d* = 0.2), medium (*d* = 0.5), and large (*d* ≥ 0.8).

### 2.6. Ethics

This study was approved by the Institutional Review Board of Louisiana State University Health Sciences Center Shreveport (IRB Study00001896; original approval 11 July 2022). Written informed consent was obtained from all participants prior to study participation. In accordance with the approved protocol and institutional policies, all assessment data and interview recordings were stored securely, and identifiable information was removed from interview transcripts to protect participant confidentiality.

## 3. Results

### 3.1. Participant Characteristics

Eleven participants from across the United States enrolled in this study (eight female, three male); however, two participants withdrew early due to unanticipated family needs. The final analytic sample therefore included nine participants (eight female, one male). All nine participants completed the training intervention and the workplace self-efficacy survey. Pre–post qualitative interviews and cognitive assessments were available for eight participants, reflecting one instance of incomplete secondary outcome data.

To protect participant privacy, ages were rounded to the nearest half-decade, and occupations were reported in general categories. The participants’ ages ranged from 20 to 55 years (M = 40.0, SD = 10.7). The participant demographics are illustrated in Table 1.

History of SARS-CoV-2 infection was ascertained via self-reporting, with participants reporting the number of confirmed positive COVID-19 tests and the approximate dates of infection. The median time between SARS-CoV-2 infection and study participation was 21 months (range = 11–76 months). None of the participants required hospitalization during their acute COVID-19 illness. At the time of enrollment, the participants reported persistent post-acute sequelae consistent with NC-PASC. The reported post-COVID-19 symptoms spanned cognitive, autonomic, and affective domains and included cognitive dysfunction (“brain fog”), impaired attention and memory, slowed processing speed, fatigue, headaches, sleep disturbances, anxiety, dysautonomia, and attention-related difficulties. Several participants also reported ongoing pulmonary manifestations, including dyspnea and reduced exercise tolerance. Four participants reported receiving ongoing care through specialized long-COVID clinics.

Total training exposure varied across participants, reflecting the adaptive delivery model of the intervention. Total training hours ranged from 45 to 90 h, with participants completing between 30 and 90 training sessions. Session duration was either 60 or 90 min, and the total training period spanned 4 to 13 months. Variability was observed in the number of sessions required to complete training as well as in the overall duration of participation. The individual training parameters are summarized in Table 2.

### 3.2. Workplace Self-Efficacy Survey Results

The primary outcome for this study was to evaluate changes in perceived workplace self-efficacy for the participants. We first assessed this outcome using the scores on the Workplace Self-Efficacy Survey (WSES) completed by the participants. We performed a within-group group repeated measures Multivariate Analysis of Variance (MANOVA) to determine whether the overall Workplace Self-Efficacy Score as well as the ratings of perceived work performance and perceived cognitive functioning at work changed over three timepoints: before COVID-19, after getting COVID-19, and after cognitive training. The overall multivariate test was significant, Wilk’s Lambda = 0.251, *F*(6,28) = 4.65, *p* = 0.002, $\eta_{p}^{2}$ = 0.50, indicating there were significant differences in the survey ratings over time with a medium effect. To illustrate where those differences lie, we report the univariate repeated measures ANOVA results below. The mean ratings on the 29 individual items on the survey are presented in Appendix A and the results of the univariate contrasts on the three composite scores are illustrated in Table 3 and described below.

#### 3.2.1. Workplace Self-Efficacy Score

A repeated measures ANOVA was used to evaluate the changes in the overall Workplace Self-Efficacy composite score over three timepoints: before COVID-19, after COVID-19, and after cognitive training. Mauchly’s Test of Sphericity revealed the assumption of sphericity was met for the repeated measures, W = 0.882, X^2^(2) = 0.878, *p* = 0.645. There was a statistically significant effect of Time on the Workplace Self-Efficacy Score, *F*(2,16) = 18.28, *p* < 0.001, $\eta_{p}^{2}$ = 0.69 with a large effect size. Post hoc testing with a Bonferroni correction revealed that the Workplace Self-Efficacy Score was significantly higher at Time 1 before getting COVID-19 (M = 239.56, SD = 4.9) than at Time 2 after getting COVID-19 (M = 119.66, SD = 22.1, *p* = 0.002). The Workplace Self-Efficacy Score was also significantly higher at Time 3 after cognitive training (M = 201.22, SD = 22.1) than it was at Time 2 after getting COVID-19 but before training (*p* = 0.003). There was no significant difference between the Workplace Self-Efficacy Score at Time 1 before getting COVID-19 and at Time 3 after training (*p* = 0.35), indicating the participants believed their function in this area after the intervention was similar to their function prior to COVID-19.

#### 3.2.2. Work Performance Rating

A repeated measures ANOVA was used to evaluate the changes in perceived Work Performance over three timepoints: before COVID-19, after COVID-19, and after cognitive training. Mauchly’s Test of Sphericity revealed the assumption of sphericity was met for the repeated measures, W = 0.843, X^2^(2) = 1.19, *p* = 0.549. There was a statistically significant effect of Time on the Work Performance rating, *F*(2,16) = 17.66, *p* < 0.001, $\eta_{p}^{2}$ = 0.69 with a large effect size. Post hoc testing with a Bonferroni correction revealed that the Work Performance rating was significantly higher at Time 1 before getting COVID-19 (M = 177.56, SD = 3.2) than at Time 2 after getting COVID-19 (M = 90.89, SD = 16.1, *p* = 0.002). The Work Performance rating was also significantly higher at Time 3 after cognitive training (M = 148.11, SD = 15.4) than it was at Time 2 after getting COVID-19 but before training (*p* = 0.003). There was no significant difference between the Work Performance Rating at Time 1 before getting COVID-19 and at Time 3 after training (*p* = 0.31), indicating the participants believed their function in this area after the intervention was similar to their function prior to COVID-19.

#### 3.2.3. Cognitive Performance Rating

A repeated measures ANOVA was used to evaluate the changes in perceived Cognitive Performance over three timepoints: before COVID-19, after COVID-19, and after cognitive training. Mauchly’s Test of Sphericity revealed the assumption of sphericity was met for the repeated measures, W = 0.987, X^2^(2) = 0.091, *p* = 0.956. There was a statistically significant effect of Time on the Cognitive Performance Rating, *F*(2,16) = 17.91, *p* < 0.001, $\eta_{p}^{2}$ = 0.69 with a large effect size. Post hoc testing with a Bonferroni correction revealed that the Cognitive Performance rating was significantly higher at Time 1 before getting COVID-19 (*M* = 62.00, *SD* = 2.1) than at Time 2 after getting COVID-19 (*M* = 28.78, *SD* = 6.3, *p* = 0.001). The Cognitive Performance rating was also significantly higher at Time 3 after cognitive training (*M* = 53.11, *SD* = 5.2) than it was at Time 2 after getting COVID-19 but before training (*p* = 0.006). There was no significant difference between the Cognitive Performance rating at Time 1 before getting COVID-19 and at Time 3 after training (*p* = 0.52), suggesting the participants believed their function in this area after the intervention was similar to their function prior to COVID-19.

#### 3.2.4. Home Functioning Rating

To evaluate the changes in perceived Home Functioning over the three time points, we analyzed the three individual items from the Home Functioning scale using the Friedman test followed by pairwise Wilcoxon signed-rank tests with Bonferroni correction to identify specific differences between the timepoints. Table 4 illustrates the mean rating, standard deviation, and the changes over time for the three perceived Home Functioning items: *Ability to maintain personal hygiene and appearance standards*, *ability to maintain home order and neatness*, and *ability to balance work and home responsibilities*. The ratings on perceived ability to maintain *personal hygiene and appearance* changed significantly across timepoints, Friedman χ^2^(2) = 12.33, *p* < 0.001. The post hoc Wilcoxon tests revealed a non-significant decline from T1 to T2 (Z = −2.37, *p* = 0.054), a significant increase from T2 to T3 (Z = −2.41, *p* = 0.048), and non-significant difference between T1 and T3 (Z = −0.816, *p* = 1.24), indicating the participants believed their function in this area after the intervention was similar to their function prior to COVID-19. The ratings on perceived ability to *maintain* *home order and neatness* changed significantly across timepoints, Friedman χ^2^(2) = 13.86, *p* < 0.001. The post hoc Wilcoxon tests revealed a significant decline from T1 to T2 (Z = −2.53, *p* = 0.033), a significant increase from T2 to T3 (Z = −2.53, *p* = 0.033), and non-significant difference between T1 and T3 (Z = −0.1.62, *p* = 0.312), indicating the participants believed their function in this area after the intervention was similar to their function prior to COVID-19. Finally, the ratings on perceived ability to *balance* *home and work responsibilities* changed significantly across timepoints, Friedman χ^2^(2) = 13.27, *p* < 0.001. The post hoc Wilcoxon tests revealed a significant decline from T1 to T2 (Z = −2.67, *p* = 0.021), a significant increase from T2 to T3 (Z = −2.52, *p* = 0.036), and non-significant difference between T1 and T3 (Z = −0.847, *p* = 1.19), indicating the participants believed their function in this area after the intervention was similar to their function prior to COVID-19.

### 3.3. Qualitative Interview Results

Of the nine participants in the study, eight completed the pre and post interviews. The qualitative thematic analysis was conducted on the qualitative data from those eight. Five main themes emerged in both the intake (pre-intervention) and exit (post-intervention) qualitative interview data: executive function, health and energy, daily living activities, productivity, and socioemotional functioning. Table 5 summarizes these main themes, the percentage of participants who reported them, and examples of the items in each theme.

#### 3.3.1. Executive Function

The theme of executive function captures the extensive cognitive challenges, specifically in relation to executive functions, following COVID-19 and the subsequent perceived cognitive and executive function improvements after the intervention. It reflects difficulties such as initiating and completing tasks, maintaining attention and focus, processing information (both verbal and visual), problem solving, planning, and memory (short- and long-term). For example, one participant lamented after COVID-19, “My thoughts were scattered, making organization difficult…my thoughts overlapped, making it hard to categorize and keep them in order.”

This theme also reflects participants’ post-training perceived improvements in memory, planning, attention, and problem solving. One participant said, “If I’m comparing now with six months ago, it’s night and day. I feel like I’m at about 80% of where I was before I got sick.” Another said, “I have more motivation sometimes,” and “I still struggle with long-term focus, but it’s better.” Other participants describe significant improvements such as, “I can figure things out [now],” “I know I wouldn’t have been able to do this in the condition I was in before,” and “Memories come back randomly and…it brings me joy when I remember something!” One participant also said, “My attention to detail, short term memory, and ability to not get distracted in loud environments is the best I can remember since before COVID.”

#### 3.3.2. Productivity

The theme of productivity addresses the perceived post-COVID-19 impact on professional, academic, and work-related activities. In the pre-intervention interviews, examples included reduced work output, diminished work quality, challenges in meeting work and/or academic deadlines, lower productivity, frequent errors, fears of career stagnation, loss of employment, and academic failure. In the post-intervention interviews, participants mentioned perceived improvements in work attendance, productivity, planning, and the ability to complete a normal workload. One participant described, “Before COVID I worked 24 h shifts and I always knew my deadlines and benchmarks. After COVID I had brain fog…my thinking slowed down, I started screwing up dates and times.” After the intervention, the same participant reported “I feel sharp again. I’m driven. I’m going back to college.” Another participant reported a dramatic improvement in grades saying, “I’m much more efficient in balancing responsibilities. I can make a plan, stick to it, and get things done…Now I can complete tasks.” Every participant reported some level of improved productivity with work or academics such as, “I stay busy and productive,” and “Now, I’m doing my job well.”

#### 3.3.3. Health and Energy

This theme reflects the widespread physical health challenges and energy constraints experienced after COVID-19, as well as the strategies participants adopted to manage these issues. At the pre-intervention interview, participants reported post-exertional malaise, lingering lethargy, nerve pain, disrupted sleep, and autonomic dysfunction (e.g., POTS, migraines). One participant mentioned significant ongoing health issues with shortness of breath, fatigue, and dizziness. The same participant discussed fatigue-related problems with confusion, inattention, and an inability to follow conversations stating, “I have no concept of time, no schedules, no thought process; I’m just trying to get through the day…I forget what I’m saying or can’t keep simple conversations.” Another mentioned, “It’s too hard to drive; I get very sleepy and can’t stay focused,” while another said, “Even social interaction drains me.” After completing the intervention, participants described developing structured sleep and exercise routines, and perceived improvements in energy resulting in more motivation, better focus, and feeling more cognitively healthy. One stated, “I stay on task, organize, declutter, and feel like myself again.”

#### 3.3.4. Daily Living Activities

This theme encompasses the post-COVID-19 challenges in the Activities of Daily Living (ADLs) and Instrumental Activities of Daily Living (IADLs), such as managing day-to-day living tasks, personal hygiene, and maintaining an orderly home, as well as the perceived post-intervention improvements in these same areas. Prior to the intervention, multiple participants described a post-COVID-19 decline in their ability to keep up with cleanliness, organization, and scheduling of personal activities. One participant stated, “It’s a struggle just to manage basic tasks,” and another said, “Mostly it’s just an inability to focus long enough to finish [household] tasks.” One said, “Initiating tasks is much harder. I procrastinate even small things like ordering pet food…I forgot to pack my child’s lunch, first time ever. Later, I couldn’t remember what day it was.” After the intervention, participants described perceived specific improvements such as, “My house isn’t like I used to keep it…but I pace myself and do better now,” “I can help my daughter with her homework again, which I couldn’t do before,” and “Now I can put household tasks down and come back to them [later]…before I couldn’t focus at all.”

#### 3.3.5. Socioemotional Functioning

This theme reflects the emotional and psychological toll of COVID-19 on confidence, personal identity, and social relationships. Prior to the intervention, participants expressed emotional distress, depression, anxiety, and feelings of isolation that affected self-confidence and interpersonal interactions saying, “I get overwhelmed really fast. I struggle with lack of motivation.” Some reported feeling overwhelmed, irritable, unmotivated, impatient, and embarrassed of struggling with memory. For some, conversation and speaking became harder: “It’s overwhelming…I get anxious about forgetting things,” and “I can’t articulate like I used to,” and “I struggle to get words out—it feels like a stutter, but it’s not a speech issue, it’s my brain not working as fast. It’s scary.” After the intervention, participants stated that motivation, confidence, memory, focus, and communication skills had improved, making statements such as “I articulate better, process information faster, and stay calm under pressure.” One participant described perceived improved social interactions saying, “I retain conversations better, I hear and understand people again…this training has been life-changing.” Other participants also claimed improved emotional and psychosocial functioning making statements such as, “I have better self-control in interactions,” “Now I can have a conversation despite what I’m feeling,” “I can follow discussions better,” and “I’m closer to my family, and I’m mentally in a better place.”

#### 3.3.6. Work and School Cross-Case Synthesis

Across the pre-intervention interviews, all eight participants who completed the interviews described substantial challenges with their ability to perform at work or school. They reported difficulties with brain fog, reduced attention spans, slow verbal processing, reading comprehension struggles, challenges learning and retaining new information, and memory deficits. They also reported issues with planning, prioritizing, time management, task initiation, frequent forgetfulness, follow-through, and even safety. Several described how exhaustion limited their ability to go to work or school or sustain a full day. They reported emotional struggles that impacted their confidence and abilities in work or school. After the intervention, participants described perceived significant improvements in these areas. Table 6 presents this cross-case synthesis in work and school subthemes from pre-intervention to post-intervention.

### 3.4. Cognitive Testing Results

**Figure 2:**
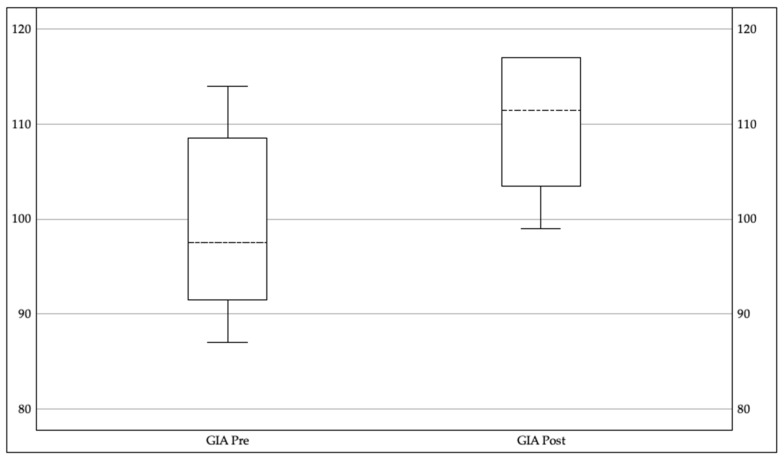
Box and whisker plot of pre- and post-GIA scores.

The secondary outcome measure for the current study was changes in overall cognitive function as measured by the Woodcock Johnson IV Tests of Cognitive Abilities. Seven subtests were administered at baseline and again at the end of cognitive training to determine changes in the participant’s overall General Intellectual Ability (GIA), or IQ score. All but one participant (who requested in-person testing) was administered the test remotely. The pretest standard scores ranged from 87 to 114 and the post-test standard scores ranged from 99 to 117. After using a paired samples *t* test on the pretest and post-test standard GIA scores, the results revealed a statistically significant improvement from pretest (*M* = 99.5, *SD* = 10.15) to post-test (*M* = 110.0, *SD* = 7.57; *t* = −5.42, *p* < 0.001, *d* = 1.9), with a mean change of 10.5 points and a large effect size. Figure 2 illustrates the pretest and post-test ranges of the scores.

## 4. Discussion

We conducted a single-arm, pilot study to evaluate pre–post changes in workplace self-efficacy and cognitive performance following a clinically and commercially available, adaptive, severity-tailored cognitive intervention in adults with post-COVID-19 brain fog. Prior research on the relevance of cognitive rehabilitation for post-COVID-19 cognitive dysfunction stressed the importance of remediating the deficits impacting daily life [29]. Unfortunately, and to our knowledge, current treatments have not led to pre-COVID-19 levels of recovery. Our study tested a cognitive training methodology which had previously shown both statistically significant and clinically significant improvements in cognition and daily functioning for populations with TBI, age-related cognitive decline, ADHD, and learning disabilities [35,36,37,38,39,40,41,42]. Therefore, our goal was to examine the feasibility and applicability of the same methodology to this population as well. Further, our mission was exploratory, hypothesis-generating, and intended to identify preliminary signals of functional improvement to inform the design of future, controlled studies rather than to establish treatment efficacy.

We chose a mixed-methods approach to this pilot examination not only to assess quantitative changes in overall workplace self-efficacy and cognition but also to capture detailed examples of the perceived changes in those same constructs elucidated by the participants. This approach produced rich data enabling us to identify clear patterns in the participants’ perceptions of change that were consistent with the changes in their overall cognitive abilities operationalized by their IQ scores.

The primary outcome of our study was workplace self-efficacy. Our analysis of the Workplace Self-Efficacy Survey indicated statistically significant changes from pre-COVID-19 to post-COVID-19 and again from pre-intervention (after getting COVID-19) to post-intervention in overall workplace self-efficacy, perceived work performance, perceived cognitive functioning, and perceived functioning at home. We also found similarities between the post-intervention ratings and the retrospective ratings of pre-COVID-19 function. While recall bias is always a risk with retrospective ratings, the participants’ perception of returning to pre-COVID-19 levels of functioning on many of the items from the survey was encouraging to note.

We wanted to explore specific examples of each participant’s experience, struggles, and perceived improvement through in-depth intake and exit interviews. As described in the Section 2, we used the Workplace Self-Efficacy Survey items as our interview guide. Our thematic analysis of the interview data revealed five main themes: executive function, health and energy, daily living activities, productivity, and socioemotional functioning. We also drilled down on the subthemes related to work and school in a cross-case synthesis of pre-intervention struggles and post-intervention improvements. The analysis of that data revealed six subthemes including cognitive processing and comprehension difficulties, memory problems impacting work or school, executive dysfunction in work or school tasks, fatigue that limited performance at work or school, emotional distress and loss of confidence in work or academics, and actual work or academic performance impairments or disruption. We noted that nearly all the participants who reported struggles in these areas after getting COVID-19 also reported improvements in these areas following the cognitive training intervention. This was consistent with our team’s prior research and with a doctoral study on adult self-efficacy whose participants reported improvements in workplace self-efficacy, achievement, leadership, emotional control, and problem solving ability after completing cognitive training with this methodology [50].

Further, to assess changes in cognition, we also administered seven subtests from a standardized cognitive skills assessment, the Woodcock Johnson IV Tests of Cognitive Abilities, to generate an overall IQ score, or GIA. Because self-reported functioning is frequently dismissed as less reliable than objective tests, we opted to add this testing to supplement our workplace self-efficacy data. Consistent with all prior research conducted by our team using this cognitive training methodology, the pretest to post-test changes in IQ score were statistically significant with a very large effect size. And although studies on other types of cognitive rehabilitation programs for post-COVID-19 cognitive dysfunction have reported significant improvements in individual cognitive constructs such as processing speed, executive function, and visual processing, this is the first study to our knowledge that documented significant changes on a traditional and widely-accepted IQ test that weights the performance across multiple cognitive constructs: working memory, long-term memory, visual processing, auditory processing, processing speed, fluid reasoning, and comprehension knowledge. We note the significant change in overall IQ score is not surprising in light of the significant change in self-reported cognitive functioning on the survey as well. Further, the comments made by participants in their post-intervention interviews are also consistent with these quantitative changes. Comments such as “My attention to detail, short term memory, and ability to not get distracted in loud environments is the best I can remember since before COVID” certainly align with these findings.

### 4.1. Study Strengths

A key strength of this study is its ecological validity, or its ability to mimic how the intervention is delivered in real-world environments. The heterogeneity of the sample and the flexibility of intervention delivery during this study help build this parallel. The heterogeneity of the sample was intentional and expected because it reflects the well-documented clinical variability in post-COVID-19 cognitive dysfunction which we highlighted in the introduction. Research is continuing to show how post-COVID-19 brain fog presents with different cognitive profiles impacting different domains such as processing speed, attention, memory, executive function, and auditory processing across age groups, severities, and comorbidities. We feel our sample parallels real-world clinical diversity which helps us evaluate the feasibility of the intervention in a heterogenous population where we see excessive individual variability.

The heterogeneity of the sample also reflects the nature of the intervention itself. The intervention is an individualized cognitive training protocol when it is used in clinical settings and learning centers. It is tailored to each person’s cognitive profile including the severity of deficits or differences in memory, attention, auditory processing, processing speed, reasoning, visual processing, and executive function strengths and weaknesses. Thus, a highly uniform sample would not have allowed us to examine how the intervention functions across a clinically diverse post-COVID-19 brain fog population and a highly uniform administration of the intervention would not have allowed us to adapt the protocols to the individual needs of the participants. Our prior work using the same methodology with children and adults who have ADHD, TBI, age-related cognitive decline, and learning disabilities has shown that individualized administration of the protocols can be implemented consistently while also accommodating participant-specific needs. In real-world settings, these protocols are adapted for each individual client’s clinical needs and scheduling constraints. Some clients attend training sessions twice per week while others have the flexibility to attend four times per week. Some clients need 60 h of training to address deficits in their cognitive profiles while others need 120 h to remediate their cognitive skill deficits. Those decisions are made in real time during the training program in collaborative discussions between the cognitive trainer, the client, and the program director. We mirrored this process in the current study. Therefore, we believe that the heterogeneity in the current sample and program delivery should be viewed as a feature of the clinical question being studied rather than a flaw in the design.

Although this was a lengthy intervention, ranging from 45 to 90 training hours over 5 to 15 months depending on the number of training sessions needed, all participants indicated the effort and time commitment had been worth it, and all but one expressed an interest to continue cognitive training past the study period. The human aspect of this intervention enables the adaptability and flexibility of the methodology without compromising the integrity or consistency of the intervention delivery. Because trainers match the intensity, complexity, and length of the program to the individual’s deficits and strengths, frustration tolerance, willingness to engage, and their work and home schedules, they were able to keep the participants in this study motivated with momentum. This practice of dynamic assessment and adaptation also mirrors the practices of cognitive trainers in the cognitive training centers where this program is delivered. Thus, the adaptive and flexible nature of the intervention delivery in this study was ecologically valid and was informed by the years of prior practice using this program delivery model. Thus, the clinical implications of this study are encouraging.

### 4.2. Study Limitations and Suggestions for Future Research

This pilot study does have several limitations, and we have suggestions for future research. First, the sample only included nine participants. Although the small sample size allowed us to examine the intervention’s potential with this population using in-depth qualitative methods, a process which is more difficult in larger samples, it limits the generalizability of the findings. Our current study’s sample size was limited by the grant funding amount, so future research should be conducted on a larger sample. Next, there is always the possibility of practice effects when you administer the same IQ test twice. That is, the participants could have improved from the first administration to the second, simply because they had practice with the test items. Although the use of standardized cognitive tests to measure progress is common in intervention research, the results of the cognitive testing should be interpreted with the possibility of practice effects in mind. Future research could mitigate this risk by using a digital cognitive test with a test bank of different items. Another limitation is the use of retrospective reporting for the baseline ratings on the survey. This introduces the possibility of recall bias, or a participant’s inability to accurately remember how they were functioning in the past. The difference between pre-COVID-19 ratings and post-intervention ratings should be interpreted accordingly. However, the significant changes in ratings from pre-intervention to post-intervention are not subject to this same recall risk and are thus encouraging and notable. Finally, this study’s design did not include a control group which limits any causal conclusions that can be made. Future research should include a control group to account for the counterfactual.

## 5. Conclusions

This mixed-methods pilot study documented the results of a Brain Strong cognitive training program for nine adults with post-COVID-19 cognitive dysfunction. The participants in this study reported improvements in workplace self-efficacy, work performance, cognition, functioning at home, executive function, health and energy, daily living activities, productivity, and socioemotional functioning. The cognitive testing results also improved. Given the small sample and non-randomized study design which limits the generalizability of this pilot work, our findings need to be interpreted cautiously. They are indeed hypothesis-generating rather than causal. Thos study supports the need for a larger, randomized controlled trial to further examine the potential benefits of Brain Strong and similar cognitive training and rehabilitation programs for post-COVID-19 cognitive sequelae and related impairments in work and school functioning.

## Figures and Tables

**Table 1:** Participant demographics.

**Age (Years)**	
20–29	1
30–39	2
40–49	4
50–59	2
Sex	
Female	8
Male	1
Race	
White	7
Black	2
Education	
Some College	4
Bachelor’s degree	2
Master’s degree	2
Doctorate degree	1
Occupation	
Legal/Personal Services	3
Military/1st Responder	2
Science and Industry	1
Writer	1
Student	2
Number of COVID-19 Occurrences at Enrollment	
One	6
Two	2
Three	1

**Table 2:** Training exposure parameters for individual participants, including total training hours, number of sessions, session duration, and overall training duration (months).

Training Hours	Number of Sessions	Session Duration (min)	Training Months
45	30	90	4
45	45	60	5
60	43	90	7
60	59	60	6
75	63	60/90	13
75	64	60	9
90	60	90	11
90	90	60	10
90	90	90	13

**Table 3:** Comparison of WSES ratings before COVID-19, after COVID-19/pre-intervention, and post-intervention.

WSES Rating	Before COVID-19 Mean (SD)	After COVID-19 Mean (SD)	Post-Intervention Mean (SD)	F	*p*	Partial Eta Squared	Pairwise Pattern
Workplace Self-Efficacy Score	239.55 (4.9)	119.66 (22.1)	201.22 (20.6)	18.3	<0.001	0.696	T1 > T2; T2 < T3; T1–T3ns
Work Performance Rating	177.56 (3.2)	90.89 (16.1)	147.11 (15.4)	17.6	<0.001	0.688	T1 > T2; T2 < T3; T1–T3ns
Cognitive Performance Rating	62.00 (2.1)	28.78 (6.3)	53.11 (5.2)	17.9	<0.001	0.691	T1 > T2; T2 < T3; T1–T3ns

Note: All pairwise comparisons were Bonferroni-corrected; T1–T3ns means no significant difference between Time 1 and Time 3 (*p* > 0.05).

**Table 4:** Comparison of individual Home Functioning ratings before COVID-19, after COVID-19/pre-intervention, and post-intervention.

Home Functioning Rating	Before COVID-19 Mean (SD)	After COVID-19 Mean (SD)	Post-Intervention Mean (SD)	T1–T2	T2–T3	T1–T3	X^2^(2)
Maintain Personal Hygiene and Appearance	9.3 (0.87)	5.9 (2.9)	8.7 (2.1)	*p* = 0.054	*p* = 0.048	*p* = 1.24	12.33 *
Maintain Home Order and Neatness	8.9 (1.5)	3.7 (3.2)	7.6 (2.6)	*p* = 0.033	*p* = 0.033	*p* = 0.312	13.86 *
Balance Home and Work Responsibilities	8.7 (1.5)	3.9 (2.1)	7.4 (3.2)	*p* = 0.021	*p* = 0.036	*p* = 1.19	13.27 *

Note: * significant at Bonferroni-corrected *p* < 0.05.

**Table 5:** Main themes of post-COVID-19 brain fog and perceived intervention impact (n = 8).

Theme	Percent Reporting	Examples of Impact
Executive Function	100%	Attention, memory, processing speed, learning, planning, time management
Health and Energy	100%	Fatigue, sleep, endurance, exercise, autonomic dysfunction
Daily Living Activities	87%	Hygiene, home organization, driving, household choirs, caregiving
Productivity	100%	Work hours, punctuality, performance, workload
Socioemotional Functioning	100%	Confidence, emotion regulation, relationships, distress

**Table 6:** Cross-case synthesis of work/school impacts and improvements.

Work/School Subtheme	Examples	Percent Reporting Difficulty Pre-Intervention	Percent Reporting Improvements Post-Intervention
Cognitive processing and comprehension difficulties	Difficulty learning new information, trouble comprehending written material, reduced attention span, slow verbal processing	100%	100%
Memory problems impacting work or school	Short-term memory lapses, forgetting tasks or appointments, needing extensive lists, difficulty retaining information	100%	88%
Executive dysfunction in work/school tasks	Procrastination, difficulty initiating or completing tasks, disorganization, poor time management, impaired planning and prioritizing	100%	100%
Fatigue limits performance	Exhaustion, mental and physical fatigue, limited ability to stay at work/school a full day	75%	63%
Emotional distress and confidence loss in work or academics	Anxiety, depression, distress about decline, loss of confidence in ability, loss of identity at work or in school roles	88%	88%
Work/academic performance impairment and disruption	Reduced productivity or accuracy, missed deadlines, underperformance, employment loss or fear of loss	100%	100%

## Data Availability

Original de-identified and de-personalized qualitative interview transcripts are available to qualified researchers upon request to the corresponding author. The de-identified quantitative dataset generated and analyzed in this study is available at Zenodo at 10.5281/zenodo.18745509.

## Abbreviations

The following abbreviations are used in this manuscript:

ADLs	Activities of Daily Living
IADLs	Instrumental Activities of Daily Living
WJIV	Woodcock Johnson IV Tests of Cognitive Abilities
GIA	General Intellectual Ability
IQ	Intelligence Quotient
WSES	Workplace Self-Efficacy Survey

## Appendix A

### Appendix A.1. Questions on Workplace Self-Efficacy Surveys

**Workplace** **Self-Efficacy Survey** **Questions**

1 being “Poor” and 10 being “Excellent”

1. Ability to maintain your personal hygiene and appearance standards
2. Ability to maintain home order or neatness
3. Ability to maintain workspace order or neatness
4. Ability to balance home and work responsibilities
5. Ability to manage time
6. Ability to make it to work on time
7. Ability to work required hours
8. Ability to plan and prioritize
9. Ability to complete simple tasks
10. Ability to accomplish larger goals
11. Problem solving ability
12. Ability to gain needed knowledge and skills
13. Initiative
14. Productivity
15. Work quality
16. Work accuracy
17. Dependability at work
18. Attention and focus
19. Patience and tolerance
20. Writing ability
21. Ability to orally communicate to peers
22. Ability to communicate to peers in writing
23. Ability to orally communicate to customers/clients
24. Ability to communicate to customers/clients in writing
25. Speed of processing verbal information
26. Speed of processing visual information
27. Speed of processing written information
28. Short-term memory
29. Long-term memory

**Scoring**

Category 1: Work Performance (Items 3, 5, 6, 7–17, 19–24)

Category 2: Cognitive Function (Items 18, 25–29)

Category 3: Home Function (Items 1, 2, 4)

Total Workplace Self-Efficacy Score = Category 1 + Category 2

### Appendix A.2. Mean Ratings Across Timepoints on Each Survey Question

**#**	**Question**	**Pre-COVID-19** **Mean** **(SD)**	**Pre-Intervention** **Mean** **(SD)**	**Post-Intervention Mean** **(SD)**
1	Ability to maintain your personal hygiene and appearance standards	9.33 (0.86)	5.89 (2.93)	8.67 (2.06)
2	Ability to maintain home order or neatness	8.89 (1.53)	3.67 (3.16)	7.56 (2.65)
3	Ability to maintain workspace order or neatness	8.56 (1.59)	4.11 (3.02)	7.33 (2.74)
4	Ability to balance home and work responsibilities	8.67 (1.50)	3.89 (2.15)	7.44 (3.17)
5	Ability to manage time	8.78 (1.20)	3.11 (2.31)	7.33 (1.41)
6	Ability to make it to work on time	8.56 (1.88)	4.67 (2.74	7.89 (2.80)
7	Ability to work required hours	9.56 (0.726	4.56 (2.74)	8.00 (3.04)
8	Ability to plan and prioritize	9.67 (0.50)	3.89 (3.22)	7.67 (2.40)
9	Ability to complete simple tasks	9.44 (0.726)	4.78 (2.59)	7.89 (2.31)
10	Ability to accomplish larger goals	8.56 (1.42)	4.00 (2.18)	7.11 (3.02)
11	Problem solving ability	9.22 (0.972)	5.11 (2.93)	8.33 (2.34)
12	Ability to gain needed knowledge and skills	9.56 (0.726)	4.78 (2.95)	8.00 (2.18)
13	Initiative	9.67 (0.50)	4.67 (2.96)	7.67 (2.45)
14	Productivity	9.11 (0.928)	3.22 (2.39)	8.00 (2.55)
15	Work quality	9.44 (0.882)	5.22 (3.31)	7.78 (3.19)
16	Work accuracy	9.56 (0.726)	5.44 (3.50)	7.67 (3.08)
17	Dependability at work	9.89 (0.333)	5.89 (3.30)	8.22 (2.91)
18	Attention and focus	7.89(1.90)	2.44 (2.24)	7.56 (2.24)
19	Patience and tolerance	8.78 (1.64)	3.89 (3.14)	7.11 (2.62)
20	Writing ability	9.78 (0.441)	5.44 (3.43)	7.89 (2.71)
21	Ability to orally communicate to peers	9.67 (0.707)	5.78(3.35)	8.56 (1.88)
22	Ability to communicate to peers in writing	9.67 (0.500)	5.67 (3.32)	8.22 (2.22)
23	Ability to orally communicate to customers/clients	9.56 (0.726)	5.56 (3.24)	8.11 (2.98)
24	Ability to communicate to customers/clients in writing	9.78 (0.833)	6.22 (3.70)	7.67 (3.12)
25	Speed of processing verbal information	8.67 (1.66)	4.22 (3.35)	7.33 (2.91)
26	Speed of processing visual information	9.78 (0.441)	4.78 (3.63)	7.89 (2.57)
27	Speed of processing written information	9.00 (1.32)	3.78 (2.54)	7.11 (2.42)
28	Short-term memory	8.22 (1.56)	3.44 (2.96)	7.22 (1.99)
29	Long-term memory	9.22 (1.09)	5.00 (3.35)	7.67 (2.066)

## References

[1] Nalbandian A., Sehgal K., Gupta A., Madhavan M.V., McGroder C., Stevens J.S., Cook J.R., Nordvig A.S., Shalev D., Sehrawat T.S. (2021). Post-Acute COVID-19 Syndrome. Nat. Med..

[2] Davis H.E., McCorkell L., Vogel J.M., Topol E.J. (2023). Long COVID: Major Findings, Mechanisms and Recommendations. Nat. Rev. Microbiol..

[3] Proal A.D., VanElzakker M.B. (2021). Long COVID or Post-Acute Sequelae of COVID-19 (PASC): An Overview of Biological Factors That May Contribute to Persistent Symptoms. Front. Microbiol..

[4] Crivelli L., Palmer K., Calandri I., Guekht A., Beghi E., Carroll W., Frontera J., García-Azorín D., Westenberg E., Winkler A.S. (2022). Changes in Cognitive Functioning after COVID-19: A Systematic Review and Meta-analysis. Alzheimers Dement..

[5] Guo P., Benito Ballesteros A., Yeung S.P., Liu R., Saha A., Curtis L., Kaser M., Haggard M.P., Cheke L.G. (2022). COVCOG 2: Cognitive and Memory Deficits in Long COVID: A Second Publication From the COVID and Cognition Study. Front. Aging Neurosci..

[6] Bonfim L.P.F., Correa T.R., Freire B.C.C., Pedroso T.M., Pereira D.N., Fernandes T.B., Kopittke L., Oliveira C.R.A.D., Teixeira A.L., Marcolino M.S. (2024). Post-COVID-19 Cognitive Symptoms in Patients Assisted by a Teleassistance Service: A Retrospective Cohort Study. Front. Public Health.

[7] Orfei M.D., Porcari D.E., D’Arcangelo S., Maggi F., Russignaga D., Ricciardi E. (2022). A New Look on Long-COVID Effects: The Functional Brain Fog Syndrome. J. Clin. Med..

[8] Theoharides T.C., Cholevas C., Polyzoidis K., Politis A. (2021). Long-COVID Syndrome-associated Brain Fog and Chemofog: Luteolin to the Rescue. BioFactors.

[9] Callan C., Ladds E., Husain L., Pattinson K., Greenhalgh T. (2022). ‘I Can’t Cope with Multiple Inputs’: A Qualitative Study of the Lived Experience of ‘Brain Fog’ after COVID-19. BMJ Open.

[10] Miller A., Song N., Sivan M., Chowdhury R., Burke M.R. (2025). Exploring the Experiences of Cognitive Symptoms in Long COVID: A Mixed-Methods Study in the UK. BMJ Open.

[11] Choutka J., Jansari V., Hornig M., Iwasaki A. (2022). Unexplained Post-Acute Infection Syndromes. Nat. Med..

[12] Meinhardt J., Radke J., Dittmayer C., Franz J., Thomas C., Mothes R., Laue M., Schneider J., Brünink S., Greuel S. (2021). Olfactory Transmucosal SARS-CoV-2 Invasion as a Port of Central Nervous System Entry in Individuals with COVID-19. Nat. Neurosci..

[13] Greene C., Connolly R., Brennan D., Laffan A., O’Keeffe E., Zaporojan L., O’Callaghan J., Thomson B., Connolly E., Argue R. (2024). Blood–Brain Barrier Disruption and Sustained Systemic Inflammation in Individuals with Long COVID-Associated Cognitive Impairment. Nat. Neurosci..

[14] Andrews M.G., Mukhtar T., Eze U.C., Simoneau C.R., Ross J., Parikshak N., Wang S., Zhou L., Koontz M., Velmeshev D. (2022). Tropism of SARS-CoV-2 for Human Cortical Astrocytes. Proc. Natl. Acad. Sci. USA.

[15] Crunfli F., Carregari V.C., Veras F.P., Silva L.S., Nogueira M.H., Antunes A.S.L.M., Vendramini P.H., Valença A.G.F., Brandão-Teles C., Zuccoli G.D.S. (2022). Morphological, Cellular, and Molecular Basis of Brain Infection in COVID-19 Patients. Proc. Natl. Acad. Sci. USA.

[16] Huang S., Fishell G. (2022). In SARS-CoV-2, Astrocytes Are in It for the Long Haul. Proc. Natl. Acad. Sci. USA.

[17] Fernández-Castañeda A., Lu P., Geraghty A.C., Song E., Lee M.-H., Wood J., O’Dea M.R., Dutton S., Shamardani K., Nwangwu K. (2022). Mild Respiratory COVID Can Cause Multi-Lineage Neural Cell and Myelin Dysregulation. Cell.

[18] Douaud G., Lee S., Alfaro-Almagro F., Arthofer C., Wang C., McCarthy P., Lange F., Andersson J.L.R., Griffanti L., Duff E. (2022). SARS-CoV-2 Is Associated with Changes in Brain Structure in UK Biobank. Nature.

[19] Turana Y., Nathaniel M., Shen R., Ali S., Aparasu R.R. (2021). Citicoline and COVID-19-Related Cognitive and Other Neurologic Complications. Brain Sci..

[20] Boldrini M., Canoll P.D., Klein R.S. (2021). How COVID-19 Affects the Brain. JAMA Psychiatry.

[21] Yong S.J. (2021). Persistent Brainstem Dysfunction in Long-COVID: A Hypothesis. ACS Chem. Neurosci..

[22] Monje M., Iwasaki A. (2022). The Neurobiology of Long COVID. Neuron.

[23] Fujimoto Y., Abe H., Eiro T., Tsugawa S., Tanaka M., Hatano M., Nakajima W., Ichijo S., Arisawa T., Takada Y. (2025). Systemic Increase of AMPA Receptors Associated with Cognitive Impairment of Long COVID. Brain Commun..

[24] Davis H.E., Assaf G.S., McCorkell L., Wei H., Low R.J., Re’em Y., Redfield S., Austin J.P., Akrami A. (2021). Characterizing Long COVID in an International Cohort: 7 Months of Symptoms and Their Impact. eClinicalMedicine.

[25] Al-Aly Z., Xie Y., Bowe B. (2021). High-Dimensional Characterization of Post-Acute Sequelae of COVID-19. Nature.

[26] Graham E.L., Clark J.R., Orban Z.S., Lim P.H., Szymanski A.L., Taylor C., DiBiase R.M., Jia D.T., Balabanov R., Ho S.U. (2021). Persistent Neurologic Symptoms and Cognitive Dysfunction in Non-hospitalized Covid-19 “Long Haulers”. Ann. Clin. Transl. Neurol..

[27] García-Molina A., García-Carmona S., Espiña-Bou M., Rodríguez-Rajo P., Sánchez-Carrión R., Enseñat-Cantallops A. (2024). Neuropsychological Rehabilitation for Post–COVID-19 Syndrome: Results of a Clinical Programme and Six-Month Follow Up. Neurol. Engl. Ed..

[28] Nagra G., Ezeugwu V.E., Bostick G.P., Branton E., Dennett L., Drake K., Durand-Moreau Q., Guptill C., Hall M., Ho C. (2025). Return-to-Work for People Living with Long COVID: A Scoping Review of Interventions and Recommendations. PLoS ONE.

[29] Braga L.W., Oliveira S.B., Moreira A.S., Martins Pereira M.E.M.D.S., Serio A.S.S., Carneiro V.D.S., Freitas L.D.F.P., Souza L.M.D.N. (2023). Long COVID Neuropsychological Follow-up: Is Cognitive Rehabilitation Relevant?. NeuroRehabilitation.

[30] Weix N.M., Shake H.M., Duran Saavedra A.F., Clingan H.E., Hernandez V.C., Johnson G.M., Hansen A.D., Collins D.M., Pryor L.E., Kitchens R. (2026). Cognitive Interventions and Rehabilitation to Address Long-COVID Symptoms: A Systematic Review. OTJR Occup. Ther. J. Res..

[31] Uswatte G., Taub E., Ball K., Mitchell B.S., Blake J.A., McKay S., Biney F., Iosipchuk O., Hempfling P., Harris E. (2026). Long COVID Brain Fog Treatment: An Early-Phase Randomized Controlled Trial of Constraint-Induced Cognitive Therapy Signals Go. Rehabil. Psychol..

[32] Bashir S., Mizrahi I., Weaver K., Fregni F., Pascual-Leone A. (2010). Assessment and Modulation of Neural Plasticity in Rehabilitation With Transcranial Magnetic Stimulation. PM&R.

[33] Lampit A., Hallock H., Valenzuela M. (2014). Computerized Cognitive Training in Cognitively Healthy Older Adults: A Systematic Review and Meta-Analysis of Effect Modifiers. PLoS Med..

[34] Gavelin H.M., Domellöf M.E., Leung I., Neely A.S., Launder N.H., Nategh L., Finke C., Lampit A. (2022). Computerized Cognitive Training in Parkinson’s Disease: A Systematic Review and Meta-Analysis. Ageing Res. Rev..

[35] Ledbetter C., Moore A.L., Mitchell T. (2017). Cognitive Effects of ThinkRx Cognitive Rehabilitation Training for Eleven Soldiers with Brain Injury: A Retrospective Chart Review. Front. Psychol..

[36] Moore A.L., Carpenter D.M., James R.L., Miller T.M., Moore J.J., Disbrow E.A., Ledbetter C.R. (2020). Neuroimaging and Neuropsychological Outcomes Following Clinician-Delivered Cognitive Training for Six Patients With Mild Brain Injury: A Multiple Case Study. Front. Hum. Neurosci..

[37] Moore A.L., Carpenter D.M., Miller T.M., Ledbetter C. (2019). ThinkRx Cognitive Training for Adults over Age 50: Clinician–Caregiver Partners in Delivery as Effective as Clinician-Only Delivery. Psychol. Neurosci..

[38] Moore A.L., Miller T.M., Ledbetter C. (2021). Remote vs. In-Person Delivery of LearningRx One-on-One Cognitive Training During the COVID-19 Pandemic: A Non-Inferiority Study. Front. Psychol..

[39] James R., Lawson Moore A., Carpenter D.M., Miller T.M., Ledbetter C. (2019). Feasibility of a Functional Medicine Approach to Slowing Clinical Cognitive Decline in Patients over Age 55: A Multiple Case Study Report. OBM Integr. Complement. Med..

[40] Moore A.L., Carpenter D.M., Miller T.M., Ledbetter C. (2018). Clinician-Delivered Cognitive Training for Children with Attention Problems: Effects on Cognition and Behavior from the ThinkRx Randomized Controlled Trial. Neuropsychiatr. Dis. Treat..

[41] Jedlicka E.J. (2017). LearningRx Cognitive Training for Children and Adolescents Ages 5–18: Effects on Academic Skills, Behavior, and Cognition. Front. Educ..

[42] Carpenter D.M., Ledbetter C., Moore A.L. (2016). LearningRx Cognitive Training Effects in Children Ages 8–14: A Randomized Controlled Trial. Appl. Cogn. Psychol..

[43] Onken L.S., Carroll K.M., Shoham V., Cuthbert B.N., Riddle M. (2014). Reenvisioning Clinical Science: Unifying the Discipline to Improve the Public Health. Clin. Psychol. Sci..

[44] Eldridge S.M., Chan C.L., Campbell M.J., Bond C.M., Hopewell S., Thabane L., Lancaster G.A. (2016). CONSORT 2010 Statement: Extension to Randomised Pilot and Feasibility Trials. BMJ.

[45] Harris P.A., Taylor R., Thielke R., Payne J., Gonzalez N., Conde J.G. (2009). Research Electronic Data Capture (REDCap)—A Metadata-Driven Methodology and Workflow Process for Providing Translational Research Informatics Support. J. Biomed. Inform..

[46] Harris P.A., Taylor R., Minor B.L., Elliott V., Fernandez M., O’Neal L., McLeod L., Delacqua G., Delacqua F., Kirby J. (2019). The REDCap Consortium: Building an International Community of Software Platform Partners. J. Biomed. Inform..

[47] Schrank F., McGrew K., Mather N. (2014). Woodcock–Johnson IV Tests of Cognitive Abilities.

[48] McGrew K., LaForte E., Schrank F. (2014). Woodcock–Johnson IV Technical Manual.

[49] Braun V., Clarke V. (2006). Using Thematic Analysis in Psychology. Qual. Res. Psychol..

[50] Highland S. (2019). Examining the Self-Efficacy Perceptions of Adults Who Completed a ThinkRx One-on-One Brain Training Program. Doctoral Dissertation.

